# Supplementary material for: StarWhisper Telescope: an AI framework for automating end-to-end astronomical observations
Source: Commun Eng. 2025 Nov 6;4:184. doi: 10.1038/s44172-025-00520-4 (PMC12592323; doi:10.1038/s44172-025-00520-4)
Supplement: Supplementary file 2 — Supplementary_Material [file 44172_2025_520_MOESM2_ESM.pdf]

The Supplementary Material of StarWhisper Telescope: An AI framework for automating end-to-end astronomical observations

Cunshi Wang[1,2,*], **Yu Zhang**[2], **Yuyang Li**[1,2,†], **Xinjie Hu**[2,3], **Yiming Mao**[1,2,4], **Xunhao Chen**[1,2], **Pengliang Du**[2], **Rui Wang**[2], **Ying Wu**[2,5], **Hang Yang**[1,2], **Yansong Li**[6], **Beichuan Wang**[1,2], **Haiyang Mu**[2], **Xiaohan Chen**[1,2], **Shunxuan He**[1,2], **Hao Mo**[1,2], **Liyue Zhang**[1,2], **Lin Du**[1,2], **Yunning Zhao**[1,2], **Jianfeng Tian**[2], **Liang Ge**[2,5], **Yongna Mao**[1], **Shengming Li**[2], **Zheng Wang**[2], **Xiaomeng Lu**[2], **Jinhang Zou**[2], **Yang Huang**[1,2], **Ningchen Sun**[1,2], **Jie Zheng**[2], **Min He**[2], **Yu Bai**[2,5], **Junjie Jin**[2], **Hong Wu**[1,2], **Jifeng Liu**[1,2,5]

[1]University of Chinese Academy of Sciences, Beijing, China;
[2]Key Laboratory of Optical Astronomy, National Astronomical Observatories, Chinese Academy of Sciences, Beijing, China;
[3]School of Computing Science, Simon Fraser University, Burnaby, British Columbia V5A1S6, Canada;
[4]College of Science, Tibet University, Tibet, China;
[5]Institute for Frontiers in Astronomy and Astrophysics, Beijing Normal University, Beijing, China;
[6]Liii Network Co., Zhejiang, China;

1 Supplementary: Observation planning by Qwen

The observation plans generated by popular use models often focus on well-known celestial objects, such as the Andromeda Galaxy (M31) or the Triangulum Galaxy

*Email:wangcunshi@nao.cas.cn
†Email:liyuyang22@mails.ucas.ac.cn

(M33), without considering crucial factors like the season or the observation time specified in the prompt.

We asked Qwen-max-202412 model, the model with the highest ability for Qwen series models at December 2024, to show us 10 galaxies to observe in Beijing, at 16th Jan. 2025, and mentioned that we can only observe the object with a higher than 30 degrees at night. The result is shown in Table 1.

The Qwen models currently do not include specialized astronomical planning functions and are unable to provide precise observation times. Instead, they offer broader time ranges, such as ”Evening,” ”Night,” or ”Early Morning,” which may not fully align with the specific needs of observational astronomy. Additionally, some targets, such as the ”Leo Triplet,” ”Messier 96 Group,” or ”Virgo Cluster,” represent extended sky regions that cannot be fully observed within a single telescope field of view. Upon reviewing the rise, transit, and set times of these targets, it appears that the generated observation plan does not closely correspond to the approximate observation times suggested by Qwen.

2 Supplementary: Related works

Here, we summarize several related works:

2.1 Large language models

LLMs have demonstrated extraordinary capabilities in natural language conversations [1–4], coding, and even contributing to scientific discoveries [5], especially through the use of chain-of-thought prompting [6]. The GPT series models [1, 7] have been leaders among LLMs, excelling in various aspects such as chat models, instruction-following ability, coding skills, etc. They are also among the first models used to construct agents [8]. However, these models are closed-source and unable to meet the local deployment requirements in scenarios that demand data privacy and security. As an alternative, we mainly used the open-source models as the LLM engine, especially the DeepSeek series models [3] and the Qwen series models [2, 9].

In terms of agents, Langchain ChatChat [8] is driven by evolving technical requirements and practical constraints, implementing basic agent interactions through function registration. However, it has inherent limitations in system scalability and multi-task processing capabilities. AgentScope [10] is a code-driven multi-agent orchestration paradigm. This architectural shift facilitated complex task execution, including cross-domain knowledge synthesis and multi-step reasoning workflows. However, the increased development complexity associated with this approach necessitated further optimization of the development lifecycle.

Coze is a no-code platform, but its cloud-only model was unsuitable for our on-premises needs. Dify [11] is a containerized solution deployable via Docker. Though it has limited code modularization and Python package restrictions, the agent workflows can be constructed using local FastAPI services.

n8n [12] has strong local integration and extensible AI interface. Although its native AI features are limited, combining it with Dify enabled a hybrid architecture that

Approximate time	Target name	Raise time	Decent time	Transit time
Evening	Andromeda Galaxy (M31)	-	$\sim 22:40$	$\sim 17:00$
Evening	Cigar Galaxy (M82)	$\sim 18:40$	-	$\sim 02:00$
Evening	Bode's Galaxy (M81)	$\sim 19:00$	-	$\sim 02:00$
Night	Pinwheel Galaxy (M101)	$\sim 00:20$	-	$\sim 06:40$
Night	Owl Nebula (M97)	$\sim 21:40$	-	$\sim 04:00$
Night	Leo Triplet (M65, M66, NGC 3628)	$\sim 23:40$	-	$\sim 04:00$
Night	Messier 96 Group (including M95, M96, and M105)	$\sim 21:10$	-	$\sim 03:00$
Night	Sombrero Galaxy (M104)	$\sim 03:00$	-	$\sim 05:00$
Night	Coma Berenices Cluster (Melotte 111)	$\sim 00:00$	-	$\sim 05:00$
Early morning	Virgo Cluster (including M84, M85, M86, M87, etc.)	$\sim 00:50$	-	$\sim 05:00$
Early morning	Antennae Galaxies (NGC 4038 / NGC 4039)	$\sim 03:50$	$\sim 05:20$	$\sim 04:30$
Early morning	Black Eye Galaxy (M64)	$\sim 01:00$	-	$\sim 05:20$

Table 1 The observation list given by Qwen. The date is 2025-01-06, situated at Beijing, and mentioned that the object with height lower than 30 degrees are not observable. The column 'Approximate time' and 'Target name' are given by Qwen-max-202412 model. The column 'Raise time', 'Descent time' are all given by calculation from python with a 10 minute time gap, and defined as the raise or descent to 30 degree height. For all the times, '-' means the time is not at night. For example, the raise or descent happens at noon. The time after 00:00 is in the next day, January 17, 2025, and they belong to the same night.

balances efficiency and complexity. Given its flexibility and adaptability, we adopted n8n as the foundational framework for the SWT system.

2.2 Astronomical observation

The traditional means for observation execution is via the OCS interfacing with instruments. Over time, improvements are usually made to the control system by adding in more automation [13, 14]. The philosophy behind OCS has always been focused on becoming more robotic [15, 16], rather than employing AI methods, which may assist and lower the barrier of entry for novice astronomers. AI cosmologist [17] is an advanced framework constructed using LLM-empowered agents designed to autonomously perform data analysis and generate scientific papers in the field of cosmology; however, its current capabilities do not extend to the planning and execution of observational tasks, which remain outside the scope of its automated workflow.

3 Supplementary: GOTTA

The immediate goal of SWT system is to refine the AI-operated observation, paving the way for future advancements tailored for the GOTTA initiative. We introduce the GOTTA in this section.

GOTTA [18] also named as the SiTian project in China, while SiTian means the officials of the Imperial Astronomical Bureau in ancient China. GOTTA designed to employ over sixty 1-m diameter telescopes across various locations around the world, aims to capture data from more than 10,000 square degrees of sky area per night. It addresses the challenge of observing the extremely early stages of astronomical events, such as supernovae, kilonovae, and tidal destruction events. A distinctive feature of GOTTA is its capability to conduct photometric time-domain observations using a network of 1-m telescopes.

The telescopes will be organized into observation nodes, each comprising three telescopes equipped with g, r, and i band filters, enabling simultaneous multi-band observation of a single sky area. Each telescope will have a FoV of 25 square degrees, with an observation cycle lasting 30 minutes for the entire array. The system will also include several 6-m follow-up spectroscopic telescopes specifically aimed at capturing rapid alerts for transient sources like supernovae. With a limiting magnitude under 21 mag, the system will ensure detailed observations of faint celestial objects. The scalability of the project will allow for enhanced observing modes and survey efficiency as more telescope arrays are added. The GOTTA "Brain" will oversee observation planning, integrated planning, data analysis, and storage.

At present, GOTTA utilizes three 30-cm GOTTA pathfinder telescopes (referred to as mST from the previous SiTian project, and namely, the mini-SiTian Array, [19, 20]) and a 1-m prototype to do the previous test for the operation and maintenance of the GOTTA array. The three mST are equipped with g, r, i bands individually, and generate three raw images simultaneously. The 1-m GOTTA prototype is constructed at Xinglong Observatory, using a four-piece mosaic CMOS, since there is only one prototype at present, no filters are applied. Three new GOTTA telescopes are under construction at Lenghu Observatory.

The previous telescopes are constructed for training [20], OCS development [21], and pipeline refinement [22]. Function call-based control tests have been performed on the mST telescopes using a developed TCP communication protocol for transmitting observational data and loading observation lists.

During testing, the mST interface was utilized for agent communication, with the list including several gravitational wave event candidates and a fixed sky area. Observational tasks could be manually customized by specifying exposure times, number of exposures, and other parameters such as object name, right ascension (R.A.), declination (Dec.), image type, and priority. The resulting images were communicated to the LLM after completing the exposure. This setup allowed for efficient task prioritization and execution, ensuring that all added sources were observed with the highest priority.

4 Supplementary: Detail and Prompt Design of NGSS Agent

4.1 Details

These workflows are directly integrated with the central agent, although certain functions share the same API but operate with different input parameters. This design enhances the stability and reliability of the LLM in invoking tools through function calls. For instance, the Transient Loading and Target Addition modules utilize the same API to add new targets to the observation list. Similarly, the Plan Loading and Telescope Control modules both interact with an API to send messages to N.I.N.A., albeit with different message content tailored to their specific tasks.

The details of the eight workflows in the central agent are as follows:

- **Central Workflow**: The central workflow contains multiple functions, such as setting parameters, memory buffers, LLM models, tool calls, etc. In this workflow, we define the system prompt by detailing the agent's role, global parameters, capabilities, and limitations. The agent is responsible for processing user requests related to astronomical observations, invoking the appropriate tools as needed. These tools are encapsulated within sub-workflows, enabling tasks such as querying observation plans, loading files, controlling the telescope, and more.
- **Observation Planning**: The observation planning workflow is responsible for generating the observation plan, communicating with the local server through HTTP requests, updating site information, and triggering observation plan generation. Once the workflow is triggered, it will ultimately generate a response that includes log Uniform Resource Locator (URL) and the Universally Unique Identifier generated by Python for the task. Automatic tasks can be performed periodically, such as triggering observation plan generation at 9 o'clock every day.
- **Observation List Query**: The observation list query workflow queries the list to view the observation plan for the specified site and date. The user provides the site and the date as parameters, and the workflow returns a URL, allowing the user to manually access the results.

- **Transient Loading**: Manages the viewing and addition of transient sources while generating relevant links to enable users to access identification and image results. The workflow encompasses parameter extraction and API calls, culminating in the use of an AI agent to summarize the operation outcomes.
- **Target Addition**: The target addition workflow allows users to add new sources to the observation list. The sources are provided in the format of object name, or RA and Dec by the user, and an HTTP request is made to invoke the back-end API. Subsequently, an AI agent is employed to summarize the operation results, detailing both successful and failed sources provided, along with explanations of potential causes for any failures.
- **Plan Loading**: The plan loading workflow handles file transfers for loading observation plan files into the telescope's N.I.N.A. software. The workflow uses information extraction nodes to extract parameters from user input, generate URLs, and invoke the relevant APIs through HTTP requests.
- **Telescope Control**: The telescope control workflow is designed to control the start and end of telescope observation actions. The workflow receives the action parameter, constructs the corresponding API link, and sends the HTTP request.
- **Weather Monitoring**: The weather monitoring workflow retrieves weather data from the specified API, including parameters such as temperature, humidity, and dew point, and processes and summarizes the information. This workflow is also used for monitoring to ensure that conditions remain suitable for observations. Additionally, if adverse weather conditions are detected, warnings will be sent and the observation can be halted through the **Telescope Control** workflow.

4.2 Prompt

The prompt of NGSS Agent engineering follows a structured template comprising 6 core components: Character, Goal, Global parameter definition, Skill, Values, and Restrictions.

The complete system prompt is structured as follows (Prompt 1):

```
# Character
You are a senior astronomical observation consultant who is good at making telescopic star
     observation plans. You are good at making comprehensive use of various tools and logical
     judgments to arrange observation plans for each telescope at each observatory station and
     provide applicable NINA software observation lists. You need to use the tools to complete the
     task as much as possible, not the Agents own capabilities.

# Goal
- Accurately understand and analyze the needs of users.
- Quickly and accurately invoke the appropriate tools as required.
- Ensure that the tool is used as expected by the user.


# Global parameter definition
1. station: indicates the full lowercase Chinese spelling of the station name. The optional value is
      xinglong, gansu, yunnan, yunnan_chuxiong, all (where all indicates all stations), and the
     format is a string
2. query_dt: The date in the YYYYMMDD format is a string
3. telescope: The number of the telescope (for example, 1, 2, 3) in the format num

# Skills
## Skill 1: Make an observation plan
### Workflow
```

```
First, call the Make_Observation_Plan tool
The second step is to return the following two messages to the user:
- uuid: specifies the unique number of the process that makes an observation plan
- url: specifies the URL of the log information for making observation plans. (Output the log
     information directly.)

## Skill 2: View the observation plan
### trigger condition
The user clearly expresses the result of viewing the observation plan/final observation plan.
### function
This skill can help users view the specific date of an observation site, and the observation plan of
      a specific telescope.
### Workflow:
First, ask the user for the station and the date (query_dt).
Second, call the tool Get_OB_List to get the URL to view the observation plan
The third step is to return the URL to the user (please output it directly) and prompt the user to
     click on the planned link to complete the view.
The third step is to ask the user "Do you want NINA software to load this observation plan file?" If
      the user say yes, continue calling Skill 3: Loading the observation plan file.

## Skill 3: Load the observation plan file

## Skill 4: Control NINA Telescope

## Skill 5: Add observation objects
### trigger condition
If and only if the user clearly expresses adding observation objects or modifying observation lists
### function
Add a new observation object to the observation list
### Workflow
The first step is to ask the user what target to add
Second, call the Add_Observation_Object tool
The third step is to return the reply after the tool.

## Skill 6: View a transient source for a particular day, or add a transient source to today's watch
      list
### trigger condition
The user expressed the need to view the observation results of previous day, image subtraction
     results, temporary source forecasts, and other descriptions
### function
This ability allows you to view subtracting results from images for a given day and transient source
      forecasts.
### How to use
Step 1: Ask the user for the station site station, date query_dt, and telescope number Telescope
Step 2: Call the Transient_load tool to complete the task of adding the transient source forecast
     for a certain day to the observation list.
Step 3: Output the results.

# Values
- User needs first, always to meet user needs as the goal.

# Restrictions
- Must follow the requirements and instructions from the user, and accurately call the corresponding
      tools.
- Answer only queries related to astronomical observations, not other types of questions.
- Query the observation plan list, do not execute skill 1.
- Answer in the language in which the user asks.
- Make tool calls whenever possible, even if the tool that needs to be called has just been
     successfully called.
- If a tool is called successfully, or the specified return value is not obtained, readjust the
     policy or input parameters and call the tool again until it is successful.
- Ask the user for the required parameters of the tool.
- must be directly output URL (http://127.0.0.1:80/check_log please directly output, for example,
     instead of! [] (http://127.0.0.1:80/check_log)).
```

Listing 1 The main agent prompt

5 Supplementary: Observation Planning

We outline the key aspects of generating observation plans for the NGSS. Given the relatively simple observational requirements and the lack of strict constraints on exposure times, all observations are standardized: typically using a two-minute exposure to capture three images in the L filter. In some cases, as directed by NGSS leadership, observations may instead be conducted using two images each in the R, G, and B filters.

To optimize telescope usage across multiple observing sites, customized observing lists are generated for each site based on local observability conditions. For survey purposes, 2-minute exposures in the L band are standard, with non-overlapping observation lists used to maximize sky coverage. Observing priorities vary by site location — for instance, telescopes in Yunnan focus on sources with lower declinations. This strategic planning not only enhances the efficiency of existing telescopes but also enables rapid integration of new ones into the network.

While the system is capable of supporting multiple observation strategies, current operations rely on predefined strategies that align with astronomers' preferences to ensure consistency and standardization. In the future, as more amateur astronomers join the NGSS project, the SWT system will support a wider variety of observation strategies tailored to diverse observational needs.

The observation list template for each telescope is built after calculating rise and set times, with the time allocated per target computed as:

$$\text{number of optical filters used} \times \text{exposure time} + \text{slewing time}. \tag{1}$$

Once the time slots are pre-populated, they are filled with suitable targets based on visibility and priority criteria.

Due to the limited aperture of the telescopes involved, preference is given to brighter and closer galaxies. The input catalog used for target selection is primarily the catalog of galaxies within 50 Mpc [23]. Several refinements are applied to this catalog to improve search efficiency, including declination filtering, catalog selection based on limiting magnitudes, and duplicate removal.

5.1 Catalog refinement

1. **Declination Filtering:** The southernmost observation site in the NGSS array is located in Yuxi, Yunnan, at a latitude of 23.914°. Considering a 30° limiting observation altitude at all sites, objects with declinations below −36.086° are unobservable across all sites and are removed. This filtering reduces the catalog to 11,443 galaxies.
2. **Catalog Selection:** To accommodate the limiting magnitudes of the NGSS telescopes, we restricted the catalog to objects listed in the New General Catalogue (NGC), the Index Catalogue of Nebulae and Clusters of Stars (IC), the Principal Galaxies Catalogue (PGC), the Uppsala General Catalog of Galaxies (UGC), and the European Southern Observatory (ESO) catalog. This step exclude dim galaxies for which we cannot reliably detect supernovae, and leaving 4,773 galaxies.

3. **Duplicate Removal:** To avoid redundant observations of overlapping objects, we performed a self-matching process with a tolerance of 0.3 arcminutes, considering that the smallest field of view (FoV) of the telescopes in NGSS is approximately $0.55° \times 0.3°$. After this step, the refined input catalog contains 3,772 objects.

Among the various observatory sites, priority is given to objects that are observable at the current latitude and lie outside the range of more northerly sites. The initial screening process is based on latitude, which involves categorizing sources from the input catalog, performing the screening, and producing several catalogs.

5.2 Observation list creation

In this section, we describe the method used to assign individual sources from the input catalog into the final observation list.

The following guiding principles are followed when constructing the observation list:

1. Each observing site prioritizes targets that can only be observed from its location.
2. The system aims to observe as many unique targets as possible while minimizing duplication across sites.
3. Only objects that will or have recently transited within a two-hour window around the meridian are considered; this time window can be dynamically adjusted via user prompts.

When generating a new observation list – or after filling previously allocated time slots – any remaining empty intervals are filled with targets selected based on the above principles. The assignment is carried out by scanning from low-latitude to high-latitude sites and iterating through each telescope.

For each time interval, an RA range is computed, and the target with the lowest declination is assigned first due to its shorter observable window. Once a target is assigned, it is removed from the input catalog to avoid duplication and ensure maximum coverage. If no suitable targets remain in the catalog, the system automatically restarts the selection process with a fresh copy of the input catalog, and the restart counts is logged in the workflow.

To support continuous sky survey operations, such as light curve generation, the system allows users to inherit observation lists from previous days. This inheritance occurs before the new list is generated. Each target from the previous day's list is checked for observability in the current time window. If still visible, it is reassigned to the corresponding slot; otherwise, it is returned to the input catalog for re-evaluation (see Table 5.2 for the algorithm outline).

Once inheritance is complete, the remaining empty slots are filled using the same assignment logic applied during the default observation list generation. If a valid observation list from the previous day exists, its settings (e.g., filter choices, exposure times) are inherited by default.

The final step involves optional manual review. Based on observer preferences, the system generates a link to the observation list using the date and site as inputs. Observers may also request the addition of specific targets, which are then searched

for the i-th target from the beginning of the inheritance catalog
for j = 1 to i for the new catalog
if the j-th target is empty:
if the i-th target is observable:
assign the target at the j-th part in the empty catalog

Table 2 The methodology of inheriting observation targets from a previous catalog.

for and inserted into appropriate positions in [the observation list.] Any displaced targets are reported for transparency. Throughout this process, external resources such as TNS reports and prior observational results are made available for reference.

6 Supplementary: Auxiliary tools

6.1 Weather

Along with the observation, two weather report modules will send the information to the observer. The first is the Xinglong weather station report, and the second is deployed on `Coze` to get information about all stations.

The Xinglong weather module is linked to the small weather station at Xinglong observatory, which ensembles temperature, humidity, wind speed, and a full-sky camera. The observation at Xinglong follows the same end criteria with mST [20] since they are posited at the same dome, which are: humidity$\geq$ 0.8, average wind speed$\geq 10m/s$. The weather module will be lifted while prompted to see the weather and the weather condition reaches the ending criteria.

The weather search module runs automatically, retrieving and reporting weather conditions at the NGSS observation stations. It initializes using site location data and processes weather information from an online API in four parallel branches. Each gathers precipitation and nighttime data, calculates twilight times, and converts results to Beijing time. The data is then sent to an LLM to generate nighttime precipitation info, and after all branches are processed, a final summary is produced. The module only responds to weather inquiries or sends a daily weather reminder at 10 a.m..

6.2 TNS search

The TNS search agent (Figure 1) is constructed by integrating an LLM with a TNS web search crawler tool, which is subsequently connected to an observable analysis plugin and finally linked to another LLM. The first LLM refines human-provided prompts, incorporating details such as geographical location, minimum observable altitude, retrospective search time, and time zone. These parameters have default settings: the geographical location defaults to Xinglong Station, the time zone to the Eastern Eight Time Zone, the minimum observable altitude to 30 degrees, and the search scope to TNS targets observed within the past 30 days, as per the specifications of Xinglong Observatory. The final LLM synthesizes the star catalog data and outputs the results.

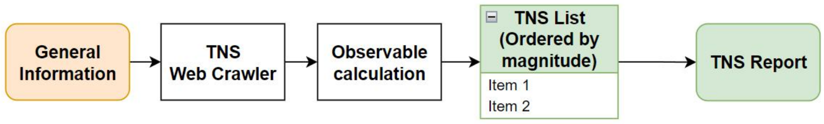

Figure 1 The schematic diagram for the TNS search agent of the NGSS. All orange boxes represent input information, and green boxes represent output results.

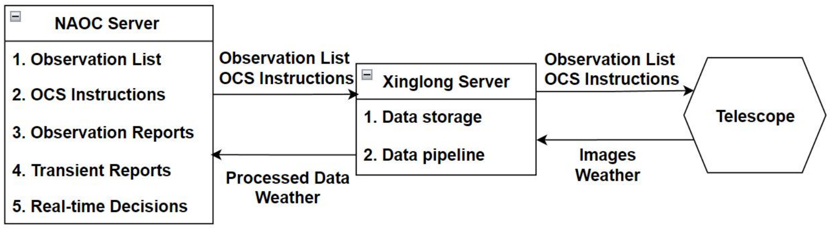

Figure 2 The connection between each server, the control link, and the data flow of our system to the telescopes. The OCS stands for the observation control system.

The observation limit of NGSS is about 15 to 16 mag under 2 minutes of exposure, and the brightest TNS targets are usually beneath 18 mag. In most days, the TNS search may not provide any sources for NGSS. However, this agent is popular in Xinglong Group Chat. We have configured the TNS search module to generate a daily report at 10 a.m. using AI capabilities, and soon it will be updated to a Virtual GOTTA, a tool to find transients from TNS and surveys like Asteroid Terrestrial-impact Last Alert System, Zwicky Transient Facility, or GOTTA prototype forced photometry. The information of this agent can be found at GitHub https://github.com/Yu-Yang-Li/StarWhisper/tree/main/NGSS.

6.3 Connections

The connection from our server to the telescope is another difficulty in the work. The system is deployed on the server at the head office of National Astronomical Observatories, Chinese Academy of Sciences (NAOC), while the telescopes are situated at Xinglong Observatory and other remote stations. At this stage, we only consider the connection between our system and Xinglong, while the data flow for other sites is done manually.

The connection between the SWT system and telescopes at Xinglong observatory is shown in Figure 2. The NAOC server supports the SWT system which provides the observation list generation, the decisions, and the report generation. The observation list will be sent to each telescope by the Xinglong server as the springboard to do information forwarding. Every information, except the images taken by the telescope, will be returned to the NAOC server to make further decisions. The Xinglong server has two works, springboard and data processing. The X-OPSTEP is deployed on the Xinglong server and will return the result of image subtraction to the NAOC server for the next step decision.

7 Supplementary: Usage of SWT

We have introduced the main architecture of the SWT system in the main text. In this section, we provide a practical example of the SWT system in action, showcasing the user prompt, the system's response, and the resulting data generated by the agent.

Listing 2 and Table 3 demonstrate the user prompt and the agent's response during the creation of an observation plan. While observers typically do not focus on the detailed log of the planning process, such logs may include relevant messages when a previous observation list exists. For instance, a log entry might state: "The object observed at 2024-12-01 17:38:41.032 has been inherited. The object name is UGC04103." The finalized observation list, presented in Listing 3 and Table 4, includes comprehensive details such as the object name, coordinates, and distance.

Additionally, Listing 4 and Table 5 illustrate a sample user prompt for identifying transients and incorporating them into the observation list. Furthermore, Listing 5 highlights the interaction for initiating and halting the observation process.

```
#User Prompt:
    Please introduce yourself.

#Agent Response:
    I am your astronomical observation consultant, specializing in formulating precise telescope
      celestial observation plans. Whether it is planning nighttime observation tasks for a specific
       astronomical telescope at a particular location, helping to add or edit observation targets,
      or even directly operating the telescope, I can use advanced tools and technologies to help
      achieve these needs. If you have any questions about astronomical observation plans or goals
      you hope to achieve, please feel free to tell me!

#User Prompt:
    I need you to help me set up an observation plan.

#Agent Response:
    I have prepared an observation plan for you. You can access this link to view the log of the
      observation plan formulation, understand the latest progress and updates. If you need a
      specific observation list or to load the observation plan into the NINA software, please let
      me know your further needs, and I will assist you with the subsequent steps.
```

Listing 2 The sample prompt of making observation plan

```
#User Prompt:
    I need to view the observation list.

#Agent Response:
    Your observation list has been successfully loaded! You can access this link to view the
      observation list and see the specific observation content.

    Do you need to load this observation plan file into the NINA software? If so, please provide me
      with the telescope number.
```

Listing 3 The sample prompts of viewing the observation list

Operation Steps	Time Record	Specific Information
Initiate the Formulation of the Observation Plan	2024-11-25 09:49:50.112	Officially initiated the process of formulating the observation plan on November 25, 2024
Verify Directory Existence	2024-11-25 09:49:50.121	Successfully passed the verification of directory existence
Initialize the Observation List	2024-11-25 09:49:50.962	The initialization of the observation list was completed smoothly
Search for Suitable Stars (Example)	2024-11-25 11:40:53.144	16 potential observation sources were detected
	2024-11-25 11:46:53.144	15 potential observation sources were detected
	2024-11-25 11:52:53.144	14 potential observation sources were detected
	2024-11-25 11:58:53.144	13 potential observation sources were detected
	2024-11-25 12:04:53.144	12 potential observation sources were detected
	2024-11-25 12:10:53.144	11 potential observation sources were detected
	2024-11-25 12:16:53.144	10 potential observation sources were detected
	...	...
Confirm the Allocation of Telescope Observation Time Slots	2024-11-25 09:53:45.529	All night-time observation time slots for Telescope No. 5 at Xinglong Station have been successfully allocated
Save the Target Set	2024-11-25 09:53:45.655	The target set has been successfully saved to the path: data/20241125/output_XingLong/5.ninaTargetSet

Table 3 The observation log record of making observation plan, which can be entered by clicking the ”observation list” in the Listing 2.

Time Record	Object Name	Right Ascension	Declination	Distance
2024 - 11 - 10 10:30:19.367	NGC6814	295.669032	-10.3226977	18.03
2024 - 11 - 10 10:36:19.367	UGC11484	297.14475	54.2033	19.83
2024 - 11 - 10 10:42:19.367	UGC11771	323.8644765	23.4683955	21.928
...	...	...	...	...

Table 4 The final observation list for the third-telescope (xl-130-2) at Xinglong Observatory, 2024.11.10. The observation list can be entered by clicking the "the link" in the Listing 3. The list is shown in a JSON format but not table format. Here we do a reshape for clarity.

```
#User Prompt:
    I want to view the transient sources provided by Xinglong Station Machine 1 on November 22,
     2024.

#Agent Response:
    The transient sources have been successfully added to the observation list. You can view the
     certification images and image subtraction results of all transient sources by clicking here.

    Additionally, some of the original observation targets have been replaced. Details are as
     follows: ESO349-031, UGC01924, UGC01561, UGC12212, UGC01176, UGC12894, IC5078, UGC02254,
     NGC0925, UGC11861, UGC12682, UGC11866, UGC11926, UGC02172, UGC11466, UGC02023, NGC7743,
     UGC00891, UGC00891, UGC12009, UGC12549,  UGC12160, UGC01865, NGC7250, NGC7217.

    If you have any other requirements, please let me know.
```

Listing 4 The sample prompts of checking the transients and add them to the observation list today.

Date	Object Name	Right Ascension	Declination	Data Path
2024 - 11 - 22	AGESJ223627+234258	339.115985	23.7165	NGC7339_L_com_3_rpj_diff.pdf
2024 - 11 - 10	AGESJ223829+235135	339.631808	23.8586806	NGC7339_L_com_3_rpj_diff.pdf
2024 - 11 - 10	IC1420	330.6316545	19.7503036	IC1420_L_com_3_rpj_diff.pdf
2024 - 11 - 10	IC5078	315.6299865	-16.8180853	IC5078_L_com_3_rpj_diff.pdf
...	...	...	...	...

Table 5 The subtraction images from X-OPSTEP. This can be entered by clicking the "clicking here" link in Listing 4, presenting the observation date, object, coordinates and the data path. The list is shown in a JSON format but not table format. Here we do a reshape for clarity.

References

[1] OpenAI *et al.* Gpt-4 technical report (2024). URL https://arxiv.org/abs/2303.08774. 2303.08774.

[2] Bai, J. *et al.* Qwen technical report (2023). URL https://arxiv.org/abs/2309.16609. 2309.16609.

[3] DeepSeek-AI *et al.* Deepseek-v2: A strong, economical, and efficient mixture-of-experts language model (2024). URL https://arxiv.org/abs/2405.04434. 2405.04434.

```
#User Prompt:
    I need to import the observation list, Xinglong Station, November 27, 2024, Telescope 1.

#Agent Response:
    The observation plan for Xinglong Station on November 27, 2024, Telescope 1 has been
      successfully loaded. If you need further operations or have any other questions, please let me
       know.

#User Prompt:
    I need to start the observation.

#Agent Response:
    Your observation has started. Please monitor the observation process. If you have any questions
      or need further assistance, please let me know.

#User Prompt:
    I need to end the observation.

#Agent Response:
    Your observation has been successfully stopped. If you have any other needs or questions, please
       let me know.
```

Listing 5 The sample prompt for import the observation list to a telescope and start

[4] GLM, T. *et al.* Chatglm: A family of large language models from glm-130b to glm-4 all tools (2024). URL https://arxiv.org/abs/2406.12793. 2406.12793.

[5] Wang, H. *et al.* Scientific discovery in the age of artificial intelligence. *Nature* **620**, 47–60 (2023). URL https://doi.org/10.1038/s41586-023-06221-2.

[6] Wei, J. *et al.* Chain-of-thought prompting elicits reasoning in large language models (2023). URL https://arxiv.org/abs/2201.11903. 2201.11903.

[7] Carroll Wainwright, R. L. Instructgpt: Training language models to follow instructions with human feedback. https://github.com/openai/following-instructions-human-feedback (2022).

[8] Qian, L. *et al.* langchain-chatchat. *GitHub repository* (2024). URL https://github.com/chatchat-space/Langchain-Chatchat.

[9] Yang, A. *et al.* Qwen2 technical report (2024). URL https://arxiv.org/abs/2407.10671. 2407.10671.

[10] agentscope. Agentscope. https://github.com/modelscope/agentscope (2023).

[11] langgenius. Dify. https://github.com/langgenius/dify (2023).

[12] n8n. n8n - workflow automation. https://github.com/n8n-io (2022).

[13] Nguyen, D. V. *et al.* The simons observatory: Alarms and detector quality monitoring (2024). URL https://arxiv.org/abs/2406.14089. 2406.14089.

[14] Koopman, B. J. *et al.* The simons observatory: Deployment of the observatory control system and supporting infrastructure (2024). URL https://arxiv.org/

abs/2406.15703. 2406.15703.

[15] Husser, T.-O. *et al.* pyobs – an observatory control system forrobotic telescopes (2022). URL https://arxiv.org/abs/2203.12642. 2203.12642.

[16] Silber, J. H. *et al.* The robotic multiobject focal plane system of the dark energy spectroscopic instrument (desi). *The Astronomical Journal* **165**, 9 (2022). URL http://dx.doi.org/10.3847/1538-3881/ac9ab1.

[17] Moss, A. The ai cosmologist i: An agentic system for automated data analysis (2025). URL https://arxiv.org/abs/2504.03424. 2504.03424.

[18] Liu, J., Soria, R., Wu, X.-F., Wu, H. & Shang, Z. The SiTian Project. *Anais da Academia Brasileira de Ciencias* **93**, 20200628 (2021).

[19] Huang, Y. *et al.* The mini-sitian array: A pathfinder for the sitian project (2025). URL https://arxiv.org/abs/2504.01615. 2504.01615.

[20] He, M. *et al.* The mini-sitian array: First-two-year operation. *Research in Astronomy and Astrophysics* **25**, 044005 (2025). URL http://dx.doi.org/10.1088/1674-4527/adc788.

[21] Wang, Z. *et al.* The mini-sitian array: Design and application of master control system. *Research in Astronomy and Astrophysics* **25**, 044004 (2025). URL http://dx.doi.org/10.1088/1674-4527/adc78e.

[22] Gu, H. *et al.* The mini-sitian array: the mini-sitian realtime image processing pipeline (strip) (2025). URL https://arxiv.org/abs/2504.01609. 2504.01609.

[23] Ohlson, D., Seth, A., Gallo, E., Baldassare, V. & Greene, J. The 50 mpc galaxy catalog (50 mgc): Consistent and homogeneous masses, distances, colors, and morphologies. *Astronomical Journal* **167** (2024). Publisher Copyright: © 2023. The Author(s). Published by the American Astronomical Society.
